# Flagellin FliC Phosphorylation Affects Type 2 Protease Secretion and Biofilm Dispersal in *Pseudomonas aeruginosa* PAO1

**DOI:** 10.1371/journal.pone.0164155

**Published:** 2016-10-04

**Authors:** Tanujaa Suriyanarayanan, Saravanan Periasamy, Miao-Hsia Lin, Yasushi Ishihama, Sanjay Swarup

**Affiliations:** 1 Metabolites Biology Lab, Department of Biological Sciences, National University of Singapore, Singapore, Singapore; 2 Microbiology Lab, Faculty of Dentistry, National University of Singapore, Singapore, Sinagpore; 3 NUS Environmental Research Institute (NERI), National University of Singapore, Singapore, Singapore; 4 Singapore Centre on Environmental Life Sciences Engineering (SCELSE), Nanyang Technological University, Singapore, Singapore; 5 Synthetic Biology for Clinical and Technological Innovation (SynCTI), Centre for Life Sciences, National University of Singapore, Singapore, Singapore; 6 Rajalakshmi Engineering College, Chennai, Tamil Nadu, India; 7 Department of Molecular and Cellular Bioanalysis, Graduate School of Pharmaceutical Sciences, Kyoto University, Kyoto, Japan; Universita degli Studi di Padova, ITALY

## Abstract

Protein phosphorylation has a major role in controlling the life-cycle and infection stages of bacteria. Proteome-wide occurrence of S/T/Y phosphorylation has been reported for many prokaryotic systems. Previously, we reported the phosphoproteome of *Pseudomonas aeruginosa* and *Pseudomonas putida*. In this study, we show the role of S/T phosphorylation of one motility protein, FliC, in regulating multiple surface-associated phenomena of *P*. *aeruginosa* PAO1. This is the first report of occurrence of phosphorylation in the flagellar protein, flagellin FliC in its highly conserved N-terminal NDO domain across several Gram negative bacteria. This phosphorylation is likely a well-regulated phenomenon as it is growth phase dependent in planktonic cells. The absence of phosphorylation in the conserved T27 and S28 residues of FliC, interestingly, did not affect swimming motility, but affected the secretome of type 2 secretion system (T2SS) and biofilm formation of PAO1. FliC phosphomutants had increased levels and activities of type 2 secretome proteins. The secretion efficiency of T2SS machinery is associated with flagellin phosphorylation. FliC phosphomutants also formed reduced biofilms at 24 h under static conditions and had delayed biofilm dispersal under dynamic flow conditions, respectively. The levels of type 2 secretome and biofilm formation under static conditions had an inverse correlation. Hence, increase in type 2 secretome levels was accompanied by reduced biofilm formation in the FliC phosphomutants. As T2SS is involved in nutrient acquisition and biofilm dispersal during survival and spread of *P*. *aeruginosa*, we propose that FliC phosphorylation has a role in ecological adaptation of this opportunistic environmental pathogen. Altogether, we found a system of phosphorylation that affects key surface related processes such as proteases secretion by T2SS, biofilm formation and dispersal.

## Introduction

Successful survival of environmental microbes in diverse niches requires highly co-ordinated switching of life strategies. The two pre-dominant lifestyles of bacteria are the free-swimming planktonic state and the surface-associated biofilm state [1, 2]. While, the planktonic state helps in active bacterial growth, biofilm state helps in bacterial survival under harsh conditions [3–6]. The development of biofilm involves five stages, namely, initial reversible attachment, irreversible attachment, microcolony formation, maturation of microcolonies to mushroom-stalk architecture, and dispersal of cells from the biofilm and return to the planktonic state [7–9].

Surface appendages such as flagella and pili can facilitate these lifestyle switches [10–12]. Among the surface-associated phenomena that are key to survival, flagella are an essential component in many Gram negative bacteria. Flagella are required for swimming, swarming, chemotaxis and biofilm formation respectively [13]. The critical role of flagella in lifestyle switch comes during biofilm attachment and detachment [9]. Flagella, which consists of multimers of flagellin, are important in the initial attachment after which flagellin is shed and in the final detachment stage, where cells again start synthesizing flagellin and return to planktonic state [14–16].

The second group of surface-associated processes that impact the bacterial lifestyle, especially during interaction with hosts are the secretion systems that affect the immediate environment. Six independent secretion systems have been classified in Gram negative bacteria, namely, Type 1–6 secretion systems [17, 18]. Some of the secretion systems exhibit cross-talk with the flagellar machinery. For instance, mutation in flagellin results in perturbations in Type 3 secretion system (T3SS) [19]. Similarly, flagellin-deficient bacteria show reduced exoproteasesof type 2 secretion system (T2SS) [20].

Post-translational modifications such as phosphorylation, glycosylation, acetylation, methylation and lipidation signal for lifestyle transition by causing structural or functional changes on fully mature proteins [21]. In recent years, S/T/Y phosphorylation has emerged as one of the major protein phosphorylation mechanisms in bacterial systems [22, 23]. Because of its ease of detection and rapid development of phosphoproteomics, S/T/Y phosphoproteome maps of *Bacillus subtilis*, *Lactococcus lactis* and *Escherichia coli* were some of the first to be estabilished [24–26]. These studies revealed association of S/T/Y phosphorylation with gene expression, transport, metabolic processes, secretion systems and virulence [22, 27, 28].

We have previously reported the phosphoproteome of *P*. *aeruginosa* PAO1, a successful environmental pathogen capable of colonizing diverse niches [29]. Among the targets identified, flagellin FliC carried phosphorylation at T27 and S28 positions respectively. The two phosphosites are present in the N-terminal NDO domain of FliC, a conserved and structurally significant region. NDO region forms the core and helps in stabilization of filament structure and assembly [14, 30]. The location of the two phosphosites in this region suggested likely biological significance. This piqued our interest to understand the different functions that this specific S/T phosphorylation of FliC might affect in *P*. *aeruginosa*. Hence, our study was directed towards the roles of FliC phosphorylation in the processes of motility, secretion and biofilm formation. Subsequently, we have uncovered effects of S/T phosphorylation of FliC on protease secretion by T2SS and biofilm dispersal. Till date, there are no other reports showing the importance of phosphorylation of a motility protein in affecting the virulence phenomena of bacteria. We have attempted to provide a novel perspective to the widespread functions of S/T/Y phosphorylation.

## Materials and Methods

### Bacterial Strains and Growth Conditions

The bacterial strains used in this study are listed in Table 1. For routine culturing, motility assays and secretome experiments, cultures were grown aerobically at 37°C in Luria-Bertani (LB) medium unless mentioned otherwise. For experiments on static biofilm and dynamic biofilm using flow cells, minimal media M9 containing 0.01% casaminoacids was used. Bacterial growth was measured spectrophotometrically at OD_600_.

**Table 1:** Bacterial strains and plasmids used in this study.

Strain/Plasmid	Characteristics	Source
PAO1 WT	Wild-type *P*. *aeruginosa* strain	[31]
Δ*fliC*	transposon library mutant of *fliC* in PAO1; Cm^r^	UW Genome Sciences library
Δ*fliC*-FL T27A	Δ*fliC* complemented with threonine phosphomutation in *fliC*; Gm^r^	This study
Δ*fliC*-FL	Δ*fliC* complemented with full length fliC; Gm^r^	This study
Δ*fliC*-FL S28A	Δ*fliC* complemented with serine phosphomutation in *fliC*; Gm^r^	This study
Δ*fliC*-FL S28D	Δ*fliC* complemented with serine phosphomimic in *fliC*; Gm^r^	This study

### Construction of FliC Phosphomutants and Phosphomimic

The phosphomutants Δ*fliC*-FL T27A and Δ*fliC*-FL S28A were generated by site directed mutation of phosphosites T27 and S28 to alanine, denoted by T27A and S28A, while the phosphomimic Δ*fliC*-FL S28D was generated by site directed mutation of phosphosite S28 to aspartate, denoted by S28D. The site-directed mutation for fliC T27A was created by fusion PCR approach, whereas, *fliC* S28A and *fliC* S28D were created by cloning full length *fliC* gene into pJET vector (Fermentas, USA), followed by mutagenesis using the Quik Change II XL Site-Directed Mutagenesis Kit (Agilent Technologies, USA). Primers used in this study are listed in Table 2. The site directed mutants and full length *fliC* gene were cloned into pUC18-miniTn7T-GM vectors and electroporated into PAO1 Δ*fliC*. The clones were verified by genomic DNA isolation and sequencing of mTn7 insertion site. The following names were used for the resulting strains: Δ*fliC*- FL T27A (complemented threonine phosphomutant), Δ*fliC*- FL (complemented full length FliC), Δ*fliC*- FL S28A (complemented serine phosphomutant) and Δ*fliC*-FL S28D (complemented serine phosphomimic).

**Table 2:** List of primers used for generating constructs.

Primers	Forward (5’-3’) sequence	Reverse (3’-5’) sequence
*fliC*	TACTGGATCCCAGACGCCAACGCCGC	CGATAAGCTTTTAGCGCAGCAGGCTCAGG
*fliC* T27A	ACGCCGCGTTGC	AAGGCGGCGTTGAGGTCG
*fliC* S28A	CCAACGACCTCAACACCGCCTTGC	GCAAGGCGGTGTTGAGGTCGTTGG
*fliC* S28D	GACCTCAACACCGACTTGCAGCGTCTG	CAGACGCTGCAAGTCGGTGTTGAGGTC
glmS	GCACATCGGCGACGTGCTCTC	CTGTGCGACTGCTGGAGCTGA
Tn7	CACAGCATAACTGGACTATTTC	ATTAGCTTACGACGCTACACCC

### Swimming Motility Assays

Plate motility assay was carried out by stabbing overnight diluted cultures in semisolid 0.3% w/v agar (BD Biosciences, USA) and determination of swim zones after 9 h and 16 h at 37°C [32]. Bacterial swim speeds were captured as follows. The cells were visualized under a 60x Plan Apo air lens (Nikon, Japan) using a Nikon Eclipse E600 microscope (Nikon, Japan). Image capture was carried out using a QICAM 12-bit CCD camera (QImaging, Canada). Cell videos were taken in AVI format for 20 s at 10 fps. At least two videos were taken per strain. Image-Pro Plus 6.3 (Media Cybernetics, USA) was used to track single cell speeds.

### Secretome Analysis

Overnight grown cultures were diluted 1:100 in 100 ml LB broth and 5 ml was harvested at approximately same OD_600_ levels after 13 h growth. Differences in growth were normalized using OD_600_ values, both for starting the cultures and at harvesting stage. Secretion starts from 9 h onwards. The supernatants were filtered through 0.20μm PES filter (Sartorius) and precipitated with Trichloroacetic acid (TCA) to get the extracellular protein profile as previously described [33]. The precipitated proteins were pelleted and washed twice with ice-cold acetone each time to remove salts and then air-dried. The solubilisation buffer was optimised to a denaturing buffer containing 40mM Tris, 40mM Dithiothreitol (DTT) and 2% SDS [34]. Densitometry analysis of protein bands from the various lanes were performed using the image analysis tool ImageJ 1.43 (http://rsbweb.nih.gov/ij/) developed by Wayne Rasband, National Institutes of Health, Bethesda, MD.

### Elastolytic Activity Assay

The assay was adapted from the method described by [35]. Elastin conjugated to Congo-red is cleaved by elastase in the extracellular protein resulting in color development and absorbance was measured at 495nm. *P*. *aeruginosa* elastase (Elastin Products Company, Inc, USA; Cat. No. PE961) was used as standard to calculate the units of active elastase per μg of total secreted protein.

### Intracellular Protein Extraction

Intracellular proteins were extracted from cell pellets after separating the supernatants for extra-cellular protein extraction. The pellets were washed once and resuspended in extraction buffer (50mM Tris, 1mM EDTA, 20mM DTT) containing complete Mini Protease Inhibitor Cocktail (Roche). Homogenization of cells was carried out in a Micro Smash MS-100 (Tomy Seiko Co., Ltd., Japan) with 0.1mm glass beads in screw cap tubes at a pulse of 4,000rpm for 20 seconds in 8 cycles. The cells were then centrifuged to get the intracellular proteins in the supernatant.

### Membrane Protein Preparation

Overnight grown cultures were diluted 1:100 in 100 ml LB broth and 50 ml was harvested at 13 h. The cell pellets were washed once with 50mM sodium phosphate buffer (pH 8) and resuspended in the same buffer containing Complete Mini Protease Inhibitor Cocktail (Roche). The cells were homogenized in a sonicator for 4 min with 40 second on and 20 second off cycle. Cell suspensions were centrifuged to remove cell debris. The supernatants were centrifuged again at 125,000g at 4°C for 30–45 minutes to separate the membrane protein fraction (pellet) and cytoplasmic and periplasmic fractions (supernatant). The pellets were dissolved in the extraction buffer with protease inhibitor and stored in—80°C until further use [36].

### Western Blot Analysis

Proteins run in 12% SDS-PAGE gel were transferred onto ECL nitrocellulose membrane to probe with respective antibodies. The XcpP, XcpY and XcpZ rabbit antibodies were kind gifts from Dr. Gerard Michel, CNRS, France [36, 37]. Mouse monoclonal antibody against alpha subunit of E. coli RNA polymerase was purchased from Neoclone Biotechnology, WI, USA and used as control. Antibodies for Las B protein and FliC protein were generated by 1^st^ base (USA) after sending them the expressed elastase B and FliC proteins as gel strips. All secondary antibodies were anti-rabbit/mouse IgG conjugated with alkaline phosphatase (Sigma). All incubations with primary and secondary antibodies were done at room temperature. The substrate used for detection was ImmobilonTM Western chemiluminescent AP substrate (Millipore).

### FliC Extraction and Phosphoprotein Analysis

Overnight cultures of one litre P. aeruginosa at early (6 h) and late (13 h) log growth phases grown at 37°C were harvested, sheared through a 23^1/2^ gauge needle and centrifuged to remove cell debris. The supernatant was centrifuged at 50,000xg for 2 h. The resultant pellets were then dissolved in acidified water (pH 2.0) adjusted with Triflouroacetic acid (TFA) and centrifuged again for 2h at 50,000xg. All buffers used contained Complete Mini Protease Inhibitor Cocktail (Roche) and Phosphatase Inhibitor (Roche) tablets. The extracted FliC was subjected to phosphorylation analysis as described below. FliC was reduced (10mM dithiothreitol) and alkylated (50 mM iodoacetamide) at room temperature. It was then digested with Lys-C (1:50, w/w) (Wako) for 3 h and then diluted 4 times with 50 mM ammonium bicarbonate for overnight trypsin (Promega) digestion at room temperature. The resulting peptide mixture was acidified with TFA and desalted with a SDB-XC solid-phase extraction cartridge. The phosphopeptides were enriched using titanium dioxide beads as described previously [38, 39]. The eluted phosphopeptides were resuspended in 0.5% TFA, 4% ACN and subjected to nanoliquid chromatography (nanoLC)—MS/MS analysis. The MS system, TripleTOF 5600, was coupled with a Ultimate 3000 RSLCnano system (Thermo Fisher Scientific) with an HTC-PAL autosampler (CTC Analytics, Zwingen, Switzerland). The MS instrument was operated in the positive ion mode, with an ion-spray voltage of 2.3 kV and an interface heater temperature of 150°C. Data were acquired from one full MS scan (*m/z* 300–1500) for 250 ms, followed by high-sensitivity MS/MS scans from the top 7 most abundant precursor ions, each with a 100-ms accumulation time.

### Phosphoprotein Data Analysis

The peak list of each raw MS spectrum was generated as previously described [40]. Peptide identification was performed by Mascot ver 2.4 (Matrix Science, London, UK) against a composite target—decoy protein sequence database containing 5,641 proteins for *P*. *aeruginosa* PAO1. The search criteria used in this study were as follows: trypsin specificity allowing up to 2 missed cleavages; fixed modification of carbamidomethyl (C); and variable modifications of oxidation (M), acetylation (protein N-term), and phosphorylation (ST) and (Y). The precursor mass tolerance was set at 20 ppm, and the fragment ion tolerance was set at 0.1 Da. Peptides were considered identified if the Mascot score yielded a confidence limit above 95% based on the significance threshold (p < 0.05) and if at least three successive y- or b-ions with an additional two and more y-, b-, and/or precursor-origin neutral loss ions were observed, based on the error-tolerant peptide sequence tag concept [41]. The phosphosites were manually checked.

### Biofilm Formation Tube Assay

The biofilm formation assay was adapted from O’ Toole and Kolter [12]. Briefly, overnight cultures grown in LB broth were diluted 1:100 and 2 ml of diluted cultures were grown in polystyrene round-bottom tubes. Cultures were incubated for 5 h at 37°C with shaking at 200 rpm. At the end of 5 h, the non-adherent cells were removed by rinsing with 5 ml of distilled water. The washed biofilms were then stained with 0.1% (w/v) crystal violet solution for 1 h followed by rinsing again with distilled water. The crystal violet bound to the tubes were solubilised by dissolving in 2ml of 1% SDS and quantitated spectrophotometrically at OD_595_.

### Image and Secretome Analysis of Static Biofilms

The imaging methods were adapted from Meritt et al. [42]. Briefly, overnight cultures grown in M9+cassamino acids media were diluted 1:100 and grown in an eight well chamber for 24 h without shaking to allow for biofilm formation. The biofilms were washed with PBS and then stained using LIVE/DEAD^®^ BacLight^™^Bacterial Viability Kit (Invitrogen, USA). Imaging was performed in Confocal Laser Scanning Microscope (LSM 780, Carl Zeiss) using the multi-track mode to minimize fluorescent bleed-through. Secretome was extracted as described previously from biofilms grown in 6-well plates under static conditions.

### Continuous-Culture Flow Cell Experiment

The flow cell experiment was adapted from [43]. Three-channel flow cells (channel dimensions, 1440 mm^3^) were used for growing biofilms [44]. The flow cells were supplied with M9 minimal medium supplemented with 0.01% cassaminoacids at 9 ml/h (mean velocity ¼0.625 mm s1) with a Reynolds number of 1.12. Each channel was injected with 0.5 ml of diluted overnight culture containing approximately 1x10^8^ cfu/ml. Biofilms were allowed to develop for 7 days and monitored every 24 h. In total, 14 flow cell setups were used for PAO1 WT, Δ*fliC*, Δ*fliC*-FL T27A, Δ*fliC*-FL and Δ*fliC*-FL S28A strains. Each day, one flow cell was sacrificed and visualized by staining with 100–200μl of live/dead stain from LIVE/DEAD^®^ BacLight^™^ Bacterial Viability Kit (Invitrogen, USA).

### Imaging and Biovolume Analysis

All microscopic observations and image acquisitions were performed by a Confocal Laser Scaning Microscope, CLSM (LSM 780, Carl Zeiss) using the multi-track mode to minimize fluorescent bleed-through. The excitation wavelengths for GFP and Ds-Red were 488 and 561 nm respectively. The emission wavelengths were 509 and 584 nm respectively. For each flow cell channel, five images near the inlet (5–10 mm from the inlet) and two images near the outlet were taken. The resultant stacks obtained from images were focussed in the centre of the channel (2 mm from the walls). For image analysis, the image stacks were quantified for each biofilm type using IMARIS (Bitplane AG, Belfast, UK). The average volume of live, dead and total cells were then calculated for estimating the attachment / detachment patterns of biofilm cells [45]. The images were represented using ortho view to represent regions of biofilm showing architecture development.

## Results

### FliC Is Phosphorylated in the N-Terminal Conserved (ND0) Domain

**Fig 1:**
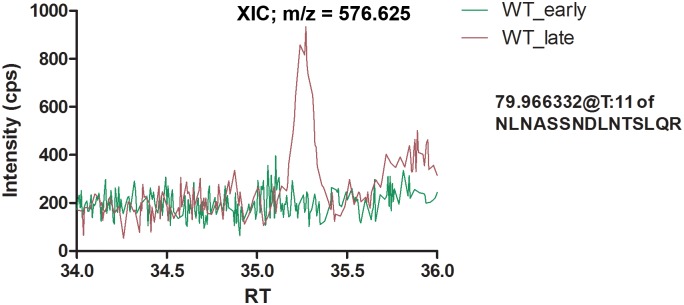
Identification of T27 phosphorylation in PAO1 FliC. Peak profile of T27 corresponding to the presence of phosphorylation in the late log phase of PAO1 WT. WT_early and WT_late denote FliC extracted from early (6 h) and late log (13 h) phase of PAO1 WT. Mass difference of 80 Da indicates phosphorylation at T27 site of FliC extracted from late log phase of PAO1 WT. Cps and RT represent counts per second and retention time respectively. Extracted ion chromatogram (XIC) denoting the mass-to-charge (m/z) ratio of the peak of interest is 576.25.

In order to confirm the presence of T27 and S28 phosphorylation in FliC, we carried out its phosphopeptide enrichment and nano LC-MS/MS from early and late log phase grown PAO1 WT. Analysis of phosphorylation profile showed T27 residue to be phosphorylated. This phosphorylation was growth-stage specific as it was present in the FliC of late log grown WT cells and not in the early log grown WT cells (Fig 1). S28 was not phosphorylated in either early or late log grown WT FliC. Though S28 was not found to be phosphorylated in this analysis, its absence in the late log FliC cannot be discounted completely because of the adjacent nature of the two phosphosites. Also, phosphomimic substitution at S28, as shown below, provided evidence of biological relevance of S28 phosphorylation of FliC.

### FliC Phosphorylation Does Not Affect Either Flagellar Motility or FliC Levels

We investigated swimming motility of PAO1 WT, Δ*fliC*, Δ*fliC*-FL T27A, Δ*fliC*-FL and Δ*fliC*-FL S28A strains by both end-point motility assay for populations and video microscopy analysis for observing speed at single cell level. The swim zones and swimming motility speeds did not have any significant differences between the phosphomutant strains and WT strains (S1 Fig). As expected, Δ*fliC* did not form any motility zone on the plate assay nor did it display any motility in the video microscopy analysis. Hence, FliC phosphorylation at T27 and S28 did not affect the swimming motility of PAO1. In order to investigate whether the loss of FliC phosphorylation affects the steady-state levels of FliC, we immunoblotted the extracted flagellin and probed with FliC antibody. The levels of FliC were similar across the WT and phosphomutant strains (S2 Fig). Together, these results show that FliC phosphorylation does not affect either flagellar motility or FliC levels.

### FliC Phosphorylation Affects T2SS

**Fig 2:**
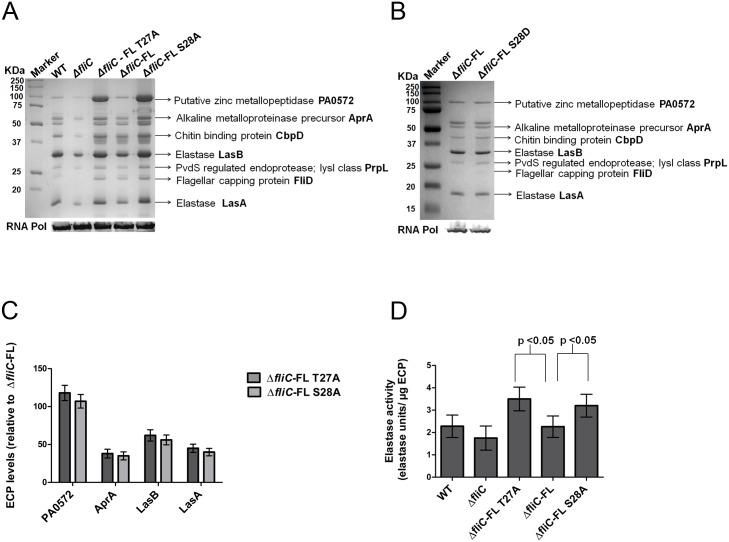
FliC phosphorylation affects type 2 secretome levels. (A& B) Total extracellular protein (ECP) from PAO1 WT, Δ*fliC*, Δ*fliC*-FL T27A, Δ*fliC*-FL and Δ*fliC*-FL S28A strains loaded based on protein secreted from equal no of cells as shown by RNA polymerase (RNA Pol) α-subunit blotting (bottom panel). (C) Quantification of proteases from (A) by Image J 1.43 software (http://rsbweb.nih.gov/ij/) showing increase in representative T2SS proteases of Δ*fliC*-FL T27A and Δ*fliC*-FL S28A. Error bars represent mean ± SD from six independent biological replicates. All differences indicated are significant with student’s t-test p-values < 0.05. (D) Elastolytic activity assay of elastase secreted by PAO1 WT, Δ*fliC*, Δ*fliC*-FL T27A, Δ*fliC*-FL and Δ*fliC*-FL S28A strains. Error bars represent mean ± SD computed from five biological replicates with student’s t-test p-values < 0.05 for Δ*fliC*-FL T27A vs. Δ*fliC*–FL and Δ*fliC*-FL S28A vs. Δ*fliC*-FL.

We investigated the effect of loss of FliC phosphorylation on the type 2 secretome profile of PAO1 WT, Δ*fliC*, Δ*fliC*-FL T27A, Δ*fliC*-FL and Δ*fliC*-FL S28A strains. We have previously profiled the type 2 secretome of PAO1 [34]. The secretome profile showed the presence of proteases such as elastases LasA & LasB, alkaline metalloproteinase AprA, chitin binding protein CbpD, endoprotease PrpL and PA0572, a zinc metallopeptidase-like protein in all five strains, consistent with previous observation. Δ*fliC* had lower levels of secretome compared to that of WT strain, consistent with previous reports [20]. WT and Δ*fliC*—FL had comparable levels of secretome. The phosphomutants of FliC, Δ*fliC*—FL T27A and Δ*fliC*—FL S28A, exhibited higher levels of secretome compared to that of WT and Δ*fliC*–FL strains (Fig 2A). The top four proteases affected by phosphomutations at both T27 and S28 were PA0572, AprA, LasB and LasA (Fig 2C). In order to ensure that the increased secretome was due to the loss of phosphorylation and not due to any structural changes, phosphomimic substitution was carried out. One of the two phosphosites, S28 was replaced with aspartate (Δ*fliC*—FL S28D), a phosphomimic. S28 was chosen because aspartate is a closer phosphomimic to serine and also to test whether it undergoes S28 phosphorylation. Secretome profile of Δ*fliC*—FL S28D was similar to that of Δ*fliC*–FL, thereby demonstrating the role of FliC phosphorylation at S28 in affecting the secretome levels of T2SS (Fig 2B). We then tested whether these increased levels of proteases led to changes in activity. Indeed, elastase activity profile of proteases was increased in Δ*fliC*- FL T27A (39%) and Δ*fliC*—FL S28A (33%) strains when compared to Δ*fliC*–FL (Fig 2D). These results suggested that FliC phosphorylation might affect T2SS functions.

### FliC Phosphorylation Selectively Affects Secretome of T2SS but Not Its Machinery Levels

**Fig 3:**
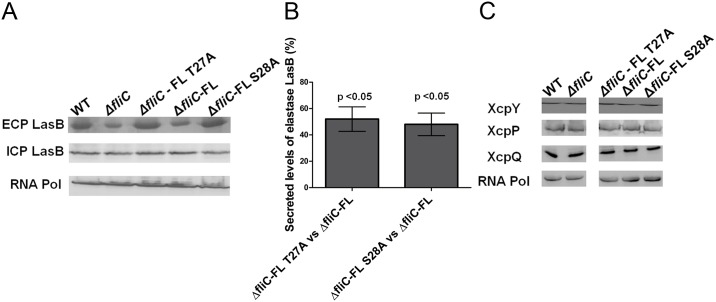
FliC phosphorylation affects T2SS secretion efficiency. (A) Immunoblot of extracellular LasB (top panel), intracellular LasB (middle panel) and intracellular RNA polymerase (RNA Pol) α-subunit (bottom panel) at 13 h for PAO1 WT, Δ*fliC*, Δ*fliC*-FL T27A, Δ*fliC*-FL and Δ*fliC*-FL S28A strains. Proteins were loaded based on equal number of cells as shown by RNA Pol α-subunit levels (bottom panel). (B) Quantification of extracellular LasB levels at 13 h in Δ*fliC*-FL T27A vs. Δ*fliC*-FL and Δ*fliC*-FL S28A vs. Δ*fliC*–FL. Error bars represent mean ±SD calculated from five biological replicates. Student’s t-test p-values < 0.05 for Δ*fliC*-FL T27A vs. Δ*fliC*–FL and Δ*fliC*-FL S28A vs. Δ*fliC*-FL. (C) Immunoblot of membrane proteins XcpY, XcpP, XcpQ in PAO1 WT, Δ*fliC*, Δ*fliC*-FL T27A, Δ*fliC*-FL and Δ*fliC*-FL S28A strains at 13 h. The proteins were loaded from equal number of bacterial cells as shown by immunoblotting of RNA Pol α-subunit levels.

In order to understand the regulation of T2SS by FliC phosphorylation, we raised the question of whether the increased secretome levels observed in the FliC phosphomutants were due to increased intracellular production of type 2 proteases or increased machinery levels of T2SS. We investigated PAO1 WT, Δ*fliC*, Δ*fliC*-FL T27A, Δ*fliC*-FL and Δ*fliC*-FL S28A strains for changes in intracellular and extracellular elastase B levels, a representative secreted protease of T2SS. Extracellular LasB showed 62% and 56% increase in Δ*fliC*—FL T27A vs. Δ*fliC*—FL and Δ*fliC*—FL S28A vs. Δ*fliC*–FL respectively, while intracellular LasB levels were unchanged (Fig 3A and 3B). This implied that the increased levels of T2SS proteases were due to increased secretion by T2SS and not due to increased translation of the intracellular proteases. To test whether T2SS machinery levels were increased, we carried out immunoblotting of representative T2SS membrane components such as XcpP, XcpY and XcpQ. The levels of membrane components of T2SS machinery were unchanged by FliC phosphorylation (Fig 3C). Together these results suggested that the observed increase in secretome could be due to control exerted at T2SS secretion efficiency.

### FliC Phosphorylation Influences Biofilm Formation under Static Conditions

**Fig 4:**
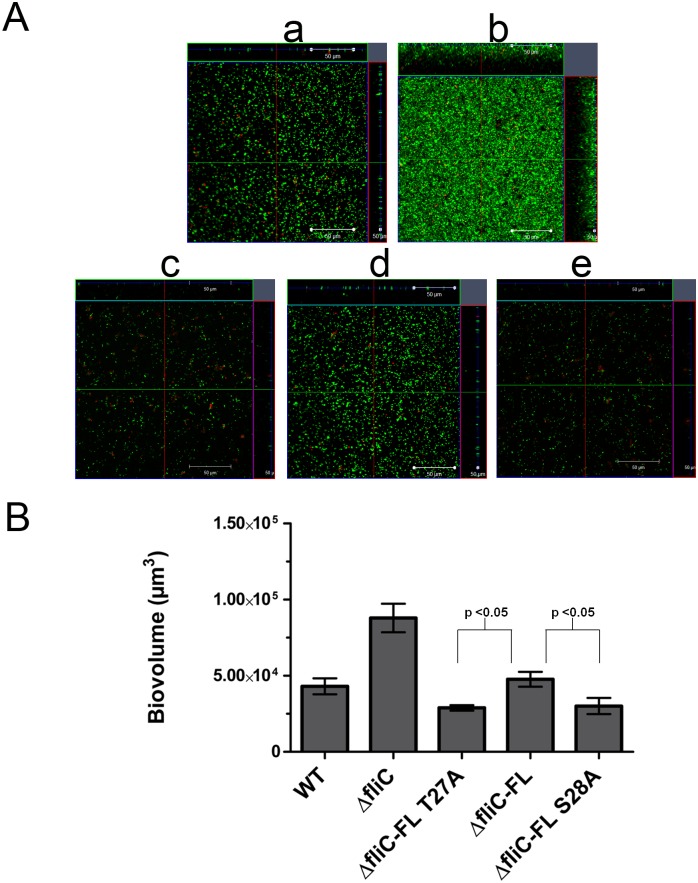
FliC phosphorylation affects static biofilm formation at 24 h. (A) Representative confocal-ortho view images of static biofilms of PAO1 WT, Δ*fliC*, Δ*fliC*-FL T27A, Δ*fliC*-FL and Δ*fliC*-FL S28A strains grown in 8 well chamber at 24 h. Live and dead cells are represented in green and red respectively. Panels are indicated as a-WT, b-Δ*fliC*, c-Δ*fliC*—FL T27A, d-Δ*fliC*–FL and e-Δ*fliC*—FL S28A, respectively. Magnification is under 40X oil lens. Scale bars indicate a distance of 50 μm. (B) Biovolumes of WT, Δ*fliC*, Δ*fliC*-FL T27A, Δ*fliC*-FL and Δ*fliC*-FL S28A strains at 24 h. Error bars indicate mean ± SD computed from five biological replicates. Student’s t-test p-values < 0.05 for Δ*fliC*-FL T27A vs. Δ*fliC*–FL and Δ*fliC*-FL S28A vs. Δ*fliC*-FL.

To determine the effect of FliC phosphorylation on biofilm formation, we performed confocal imaging of biofilms of PAO1 WT, Δ*fliC*, Δ*fliC*-FL T27A, Δ*fliC*-FL and Δ*fliC*-FL S28A strains grown under static conditions for 24 h and calculated biovolumes per unit base area. Biovolumes were reduced by 65% and 58% in Δ*fliC*- FL T27A vs. Δ*fliC*- FL and Δ*fliC*- FL S28A vs. Δ*fliC*- FL respectively (Fig 4A and 4B). The non-motile strain Δ*fliC* retained the ability to form biofilms on a glass chamber and had more attached cells than the other strains, indicating compensatory mechanisms for attachment. These results indicated that FliC phosphorylation influenced biofilm formation and the same trend was corroborated by an independent tube biofilm formation assay (S3 Fig). However, the biofilm levels of Δ*fliC* were lower in the tube biofilm assay compared to that of static biofilm assay. This type of reduced biofilm formation for flagellar mutants in tube biofilm formation assay is consistent with the previous reports on *P*. *aeruginosa* and *Salmonella typhimurium* [12, 46].

### FliC Phosphorylation Affects Secretome and Biovolume in an Inverse Manner in Static Biofilm

**Fig 5:**
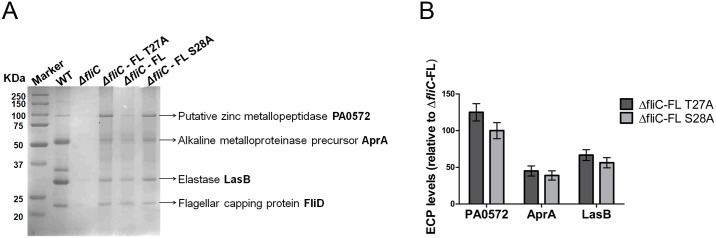
FliC phosphorylation affects type 2 secretome levels in static biofilms. (A) Type 2 secretome analysis for static biofilms of PAO1 WT, Δ*fliC*, Δ*fliC*-FL T27A, Δ*fliC*-FL and Δ*fliC*-FL S28A strains grown in 6-well plate for 24 h. Experiment was conducted with three biological replicates and two technical replicates each. (B) Quantification of proteases from (A) by Image J 1.43 software (http://rsbweb.nih.gov/ij/) showing increase in representative T2SS proteases of Δ*fliC*—FL T27A and Δ*fliC*—FL S28A. All differences are significant with student’s t-test p-values < 0.05.

In order to understand whether the effects of FliC phosphorylation on T2SS and biofilm formation are inter-related, we analysed the secretome profile of PAO1 WT, Δ*fliC*, Δ*fliC*-FL T27A, Δ*fliC*-FL and Δ*fliC*-FL S28A strains grown under static biofilm conditions. Secretome profile of the different strains contained proteases such as elastase LasB, alkaline metalloproteinase AprA and putative zinc metallopeptidase PA0572. Levels of some of the proteases were too faint to be visualized. At 24 h, Δ*fliC* had very less secretome consistent with previous observation and the two phosphomutant strains had increased secretome profile when compared to that of Δ*fliC*- FL (Fig 5A) [20]. Proteases with increased levels in the FliC phosphomutants were quantitated (Fig 5B). As shown above, at 24h, FliC phosphomutants have reduced biofilm when compared to that of Δ*fliC*- FL (Fig 4). These results indicated that the secretome profile of T2SS and biofilm formation are inversely associated.

### FliC Phosphorylation Affects Dispersal Pattern of Biofilms under Dynamic Flow Conditions

**Fig 6:**
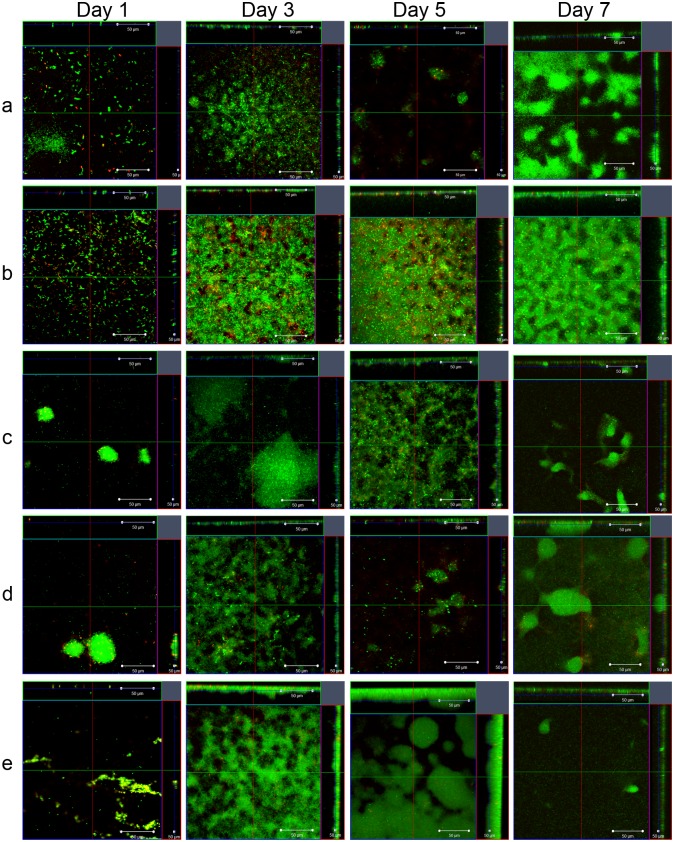
Influence of FliC phosphorylation on dynamic biofilms formed under flow cell conditions. Comparison of biofilm architecture in confocal-ortho view for PAO1 WT, Δ*fliC*, Δ*fliC*-FL T27A, Δ*fliC*-FL and Δ*fliC*-FL S28A strains across 7 days. Live and dead cells are represented in green and red, respectively. Panels are represented as a-WT, b-Δ*fliC*, c-Δ*fliC*- FL T27A, d-Δ*fliC*-FL and e-Δ*fliC*-FL S28A, respectively. Magnification is under 40X oil lens. Scale bars indicate a distance of 50 μm.

To further investigate the effect of FliC phosphorylation on biofilm formation, we studied biofilms of PAO1 WT, Δ*fliC*, Δ*fliC*-FL T27A, Δ*fliC*-FL and Δ*fliC*-FL S28A strains grown under dynamic conditions in a flow cell over a 7-day period. The typical mushroom shape like architecture developed for WT, Δ*fliC*- FL T27A, Δ*fliC-* FL and Δ*fliC*—FL S28A at different stages based on visual observation of the images. The development of architecture started from day 2 onwards, when small microcolonies began to develop. This was more obvious in Δ*fliC*- FL T27A, Δ*fliC*- FL and Δ*fliC*- FL S28A. On day 3, microcolonies formed in WT. At this stage, onset of maturation was observed in some parts of the biofilm for all strains except for Δ*fliC*. On days 4 and 5, both microcolonies formed and mature architecture began to develop, whereas by days 6 and 7, biofilm was fully developed in all strains except for Δ*fliC*. The amount of biofilm formed varied for the different strains. For WT and Δ*fliC*- FL, visual analysis of the images indicated that their biofilms increased till day 4, followed by a reduction on day 5 and then reattachment again on day 6 and day 7. For Δ*fliC*-FL T27A and Δ*fliC*- FL S28A, their biofilm increased till day 6, followed by a reduction on day 7. As reported previously, Δ*fliC* did not show development of any architecture and maintained a relatively constant level of cells throughout the 7-day experiment (Fig 6 and S4 Fig) [16].

**Fig 7:**
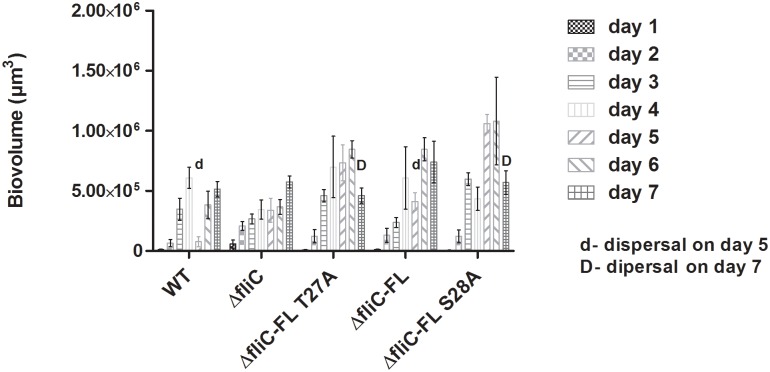
FliC phosphomutants have delayed dispersal. Total biovolumes of PAO1 WT, Δ*fliC*, Δ*fliC*-FL T27A, Δ*fliC*-FL and Δ*fliC*-FL S28A strains measured over a 7 day flow cell experiment. Reduction in biovolume from day 4 to day 5 in WT and Δ*fliC*- FL and from day 6 to day 7 in Δ*fliC*- FL T27A and Δ*fliC*- FL S28A is observed. Error bars represent mean ± SD for three biological replicates.

We quantified the biovolumes per unit base area for the 5 strains for all 7 days, which were concordant with the imaging data from Fig 6 and S4 Fig. The results showed reduction in total biovolume per unit base area on day 5 for WT and Δ*fliC*- FL, with an increasing trend till day 4. Δ*fliC*- FL T27A and Δ*fliC*- FL S28A showed reduction in total biovolume per unit base area on day 7, with an increasing trend till day 6 (Fig 7). The non-motile strain Δ*fliC* did not show any characteristic dispersal pattern. The dispersal pattern for WT and Δ*fliC*- FL corresponded with existing literature, which showed that in PAO1, dispersal occurred on day 5 followed by reattachment on day 6 and day 7 [43, 47]. The dispersal pattern for Δ*fliC*- FL T27A and Δ*fliC*- FL S28A is delayed when compared to that of WT and Δ*fliC*- FL. Results from flow cell study, therefore, showed that FliC phosphorylation can affect the dispersal pattern of biofilms.

## Discussion

S/T/Y phosphorylation has been described at proteome-wide level for a number of species across both Gram positive and negative bacteria [22, 24–27, 29, 48, 49]. While up to 100 phosphosites have been identified in some studies, no biological roles have been systematically studied for any of the newly identified phosphosites. The previously reported S/T/Y phosphorylation of kinases and proteins were characterized using hypothesis-driven approaches such as for Ppka, a threonine kinase driving the assembly of T6SS and secretion of T6SS proteases [50]. Our study has focused on the outcomes of S/T phosphorylation of flagellin FliC on its different functions such as motility, virulence, adhesion and dispersion of biofilms.

The most direct hypothesis of our study was that FliC phosphorylation affects the motility of *P*. *aeruginosa* PAO1. This hypothesis was based on the location of S/T phosphosites in the conserved N-terminal NDO domain of the FliC filament, as this domain is involved in the assembly and integrity of flagellar filaments [14, 30]. However, this hypothesis failed as swimming motility is not affected due to T27/S28 phosphorylation of FliC.

The threonine and serine phosphosites at positions 27 and 28 are conserved in the N-terminal domain of flagellin across *Pseudomonas sp*., *Escherichia sp*. and *Salmonella sp*. with the canonical sequence being (T/S)27, (T/S/A)28 (S5 Fig). This conservation of residues implied that flagellin FliC might have some function other than motility, where these phosphosites could play a role.

In the context of non-motility related functions of flagella, export machineries of flagella and secretion systems of Gram-negative bacteria are evolutionarily conserved [18, 19, 51–53]. Apart from conservation of export mechanism, some secretion systems are co-localized with the flagellar machinery. For example, T2SS and flagella are both polar localized in *P*. *aeruginosa* [54]. Some evidence of cross-talk between flagellar machinery and T2SS is seen by the effect of flagellin loss, which leads to reduced levels of proteases secreted by T2SS [20]. Hence, we tested our revised hypothesis that phosphorylation of FliC affects T2SS.

Three lines of evidence establish the effect of S/T phosphorylation on T2SS. First, levels of extracellular proteases secreted by T2SS are increased in the FliC phosphomutants and are restored in the FliC phosphomimic mutants. This highlights the role of FliC phosphomutants, especially S28 residue in T2SS functions. Second, the increase in extracellular protease levels of phosphomutants was not due to increased production of proteases, as there was no increase in intracellular levels of proteases in phosphomutants, even under increased extracellular proteases. These findings raise the possibility of a feedback mechanism that maintains the level of intracellular proteases in cells. Third, the regulation of T2SS by FliC phosphorylation did not lead to increase in secretion machinery as the levels of T2SS membrane components were unaffected by FliC phosphorylation.

Our study on the effects of FliC phosphorylation on biofilm attachment and dispersal led to two conclusions. Both initial attachment and detachment during dispersal stage were delayed by the loss of FliC phosphorylation in static and dynamic flow biofilms. As each of these processes still proceeded in the lack of phosphorylation, it suggested that FliC phosphorylation regulates the timing and rate of these processes without affecting biofilm architecture. Interestingly, in static biofilms, FliC phosphorylation seemed to be required for rapid attachment and maintaining low levels of T2SS proteases. It is possible that the association of T2SS factors in the biofilm matrix leads to decreased effectors in the culture filtrates. Hence, the FliC-T2SS interaction is also evident during biofilm growth. Overall, it is probable that FliC phosphorylation here could act to integrate environmental cues with the signals for attachment or dispersal of biofilms. FliC phosphorylation also seems to affect the impact of bacterial cells on the immediate environment through the modulation of protease secretion, hence playing a role in this two-way integration of cellular processes with the environment.

This study adds a new facet of serine and threonine phosphorylation in bacterial systems. FliC phosphorylation seems to affect both secretome levels and biofilm-related phenomena, which brings up the level of importance of FliC phosphorylation in these processes. Altogether, our study shows the presence of a phenomenon which affects at least two surface-associated processes of secretion and biofilm formation in *P*. *aeruginosa* upon FliC phosphorylation.

## Supporting Information

S1 Fig FliC phosphorylation does not affect either end point or real-time motility. (A) Quantification of motility zones formed in semisolid agar (0.3%) at 9h and16h respectively. Error bars indicate mean ± SD computed from four biological replicates with three technical replicates each. Student’s t-test p-values > 0.05 for Δ*fliC*-FL T27A vs. Δ*fliC*-FL and Δ*fliC*-FL S28A vs. Δ*fliC*-FL. at both 9h and 16h. (B) Motility speeds determined by video microscopy analysis represented as fraction of live speeds of cells falling within the different percentile categories. Error bars indicate mean ± SD computed from four biological replicates with three technical replicates each. Bonferroni multiple comparison analysis not significant, p-values > 0.05 for Δ*fliC*-FL T27A vs. Δ*fliC*-FL and Δ*fliC*-FL S28A vs. Δ*fliC*-FL.
(TIF)

S2 Fig FliC phosphorylation does not affect FliC steady-state levels. Immunoblot of extracellular FliC (top panel), and intracellular RNA polymerase (RNA Pol) α-subunit (bottom panel) at 13 h for PAO1 WT, Δ*fliC*-FL T27A, Δ*fliC*-FL and Δ*fliC*-FL S28A strains. Proteins were loaded based on equal number of cells as shown by RNA Pol α-subunit levels (bottom panel).
(TIF)

S3 Fig FliC phosphorylation affects biofilm formation at 5 h. Quantification of biofilms formed in polystyrene round-bottom tubes by crystal violet staining at 5h for PAO1 WT, Δ*fliC*, Δ*fliC*-FL T27A, Δ*fliC*-FL and Δ*fliC*-FL S28A strains. Error bars indicate mean ± SD computed from four biological replicates with five technical replicates each. Student’s t-test p-values <0.05 for Δ*fliC*-FL T27A vs. Δ*fliC*-FL and Δ*fliC*-FL S28A vs. Δ*fliC*-FL.
(TIF)

S4 Fig FliC phosphorylation affects dispersal of dynamic biofilms formed in flow cells. Comparison of biofilm architecture in confocal-ortho view for PAO1 WT, Δ*fliC*, Δ*fliC*-FL T27A, Δ*fliC*-FL and Δ*fliC*-FL S28A strains across all 7 days. Live and dead cells are represented in green and red, respectively. Panels are represented as a-WT, b-Δ*fliC*, c-Δ*fliC*- FL T27A, d-Δ*fliC*-FL and e-Δ*fliC*-FL S28A, respectively. Magnification is under 40X oil lens. Scale bars indicate a distance of 50 μm.
(TIF)

S5 Fig Multiple sequence alignment of N-terminal conserved ND0 domain of FliC. Clustal Omega alignment shows the extent of conservation of threonine 27 and serine 28 residues across different bacterial species. Species are indicated as 1- *P*. *aeruginosa* PAO1, 2- *P*. *putida* GB-1, 3- *P*. *putida* KT2440, 4- *P*. *putida* W619, 5- *P*. *putida* F1, 6- *P*. *protegens* Pf-5, 7- *P*. *flectens* Pf101, 8- *E*. *coli* K12, 9- *E*.*coli* K12 W3110, 10- *E*.*coli* CFT073, 11- *E*.*coli* EDL93 and 12- *S*. *paratyphi* A SARB42 respectively. Identical, strongly similar and weakly similar residue positions are indicated as (*), (:) and (.) respectively.
(TIF)
