# Supplementary figures and images for: Flagellin FliC Phosphorylation Affects Type 2 Protease Secretion and Biofilm Dispersal in Pseudomonas aeruginosa PAO1
Source: PLoS One. 2016 Oct 4;11(10):e0164155. doi: 10.1371/journal.pone.0164155 (PMC5049796; doi:10.1371/journal.pone.0164155)

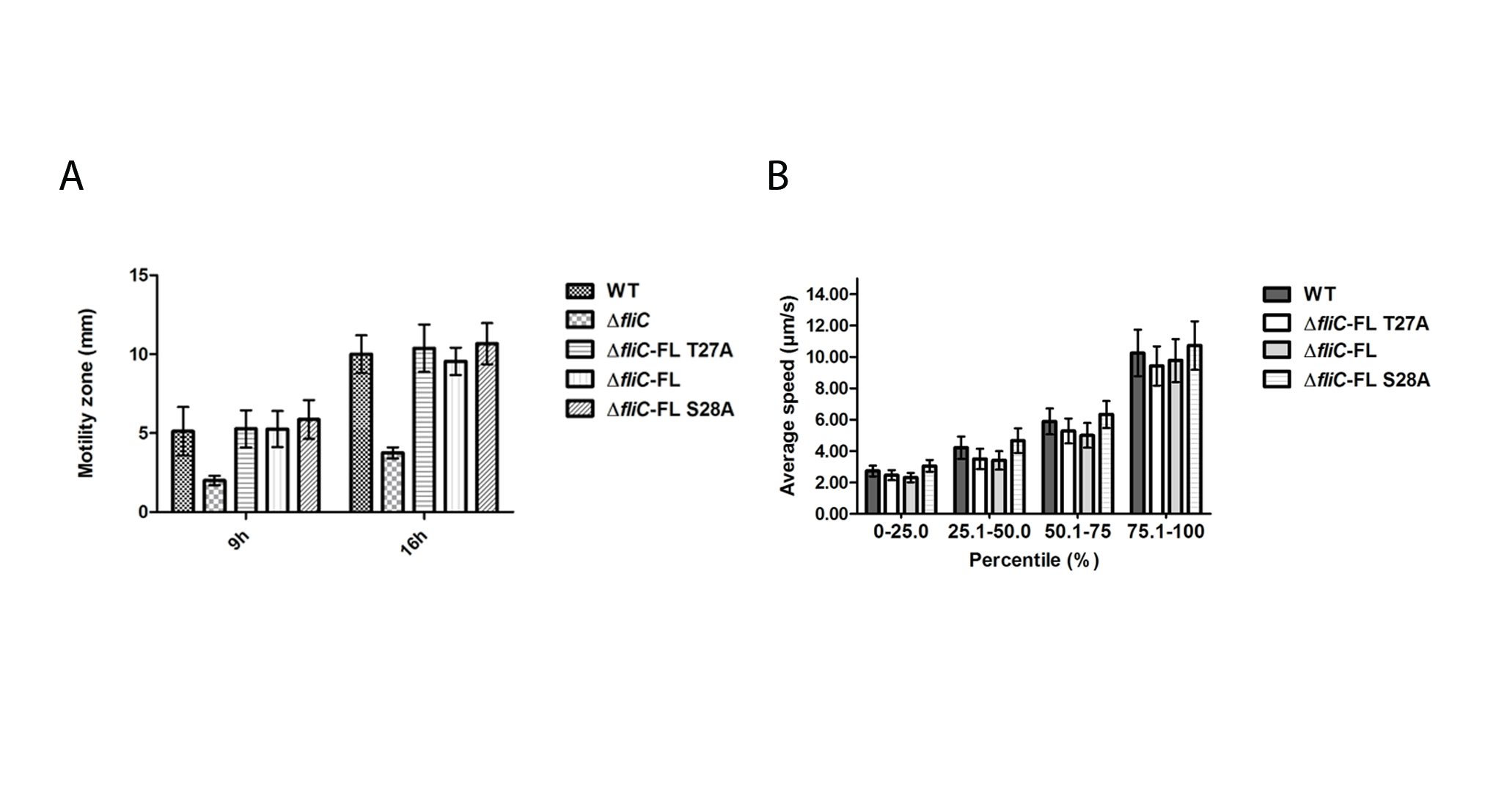

Supplement: S1 Fig — (A) Quantification of motility zones formed in semisolid agar (0.3%) at 9h and16h respectively. Error bars indicate mean ± SD computed from four biological replicates with three technical replicates each. Student’s t-test p-values > 0.05 for ΔfliC-FL T27A vs. ΔfliC-FL and ΔfliC-FL S28A vs. ΔfliC-FL. at both 9h and 16h. (B) Motility speeds determined by video microscopy analysis represented as fraction of live speeds of cells falling within the different percentile categories. Error bars indicate mean ± SD computed from four biological replicates with three technical replicates each. Bonferroni multiple comparison analysis not significant, p-values > 0.05 for ΔfliC-FL T27A vs. ΔfliC-FL and ΔfliC-FL S28A vs. ΔfliC-FL. (TIF) [file pone.0164155.s001.tif]

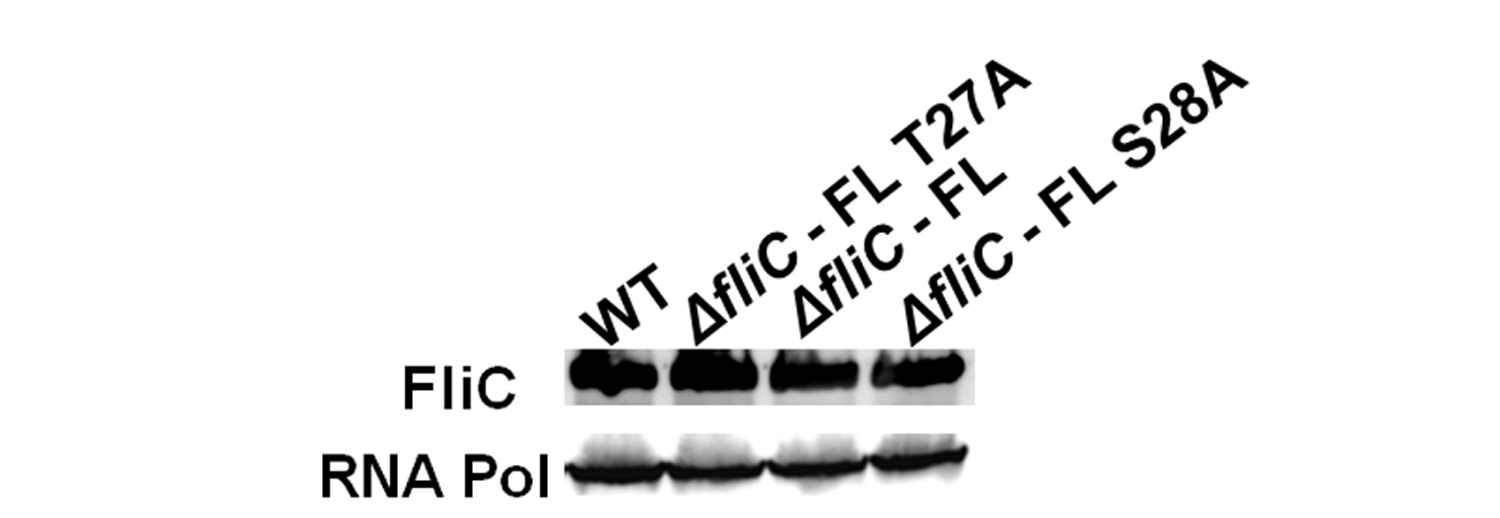

Supplement: S2 Fig — Immunoblot of extracellular FliC (top panel), and intracellular RNA polymerase (RNA Pol) α-subunit (bottom panel) at 13 h for PAO1 WT, ΔfliC-FL T27A, ΔfliC-FL and ΔfliC-FL S28A strains. Proteins were loaded based on equal number of cells as shown by RNA Pol α-subunit levels (bottom panel). (TIF) [file pone.0164155.s002.tif]

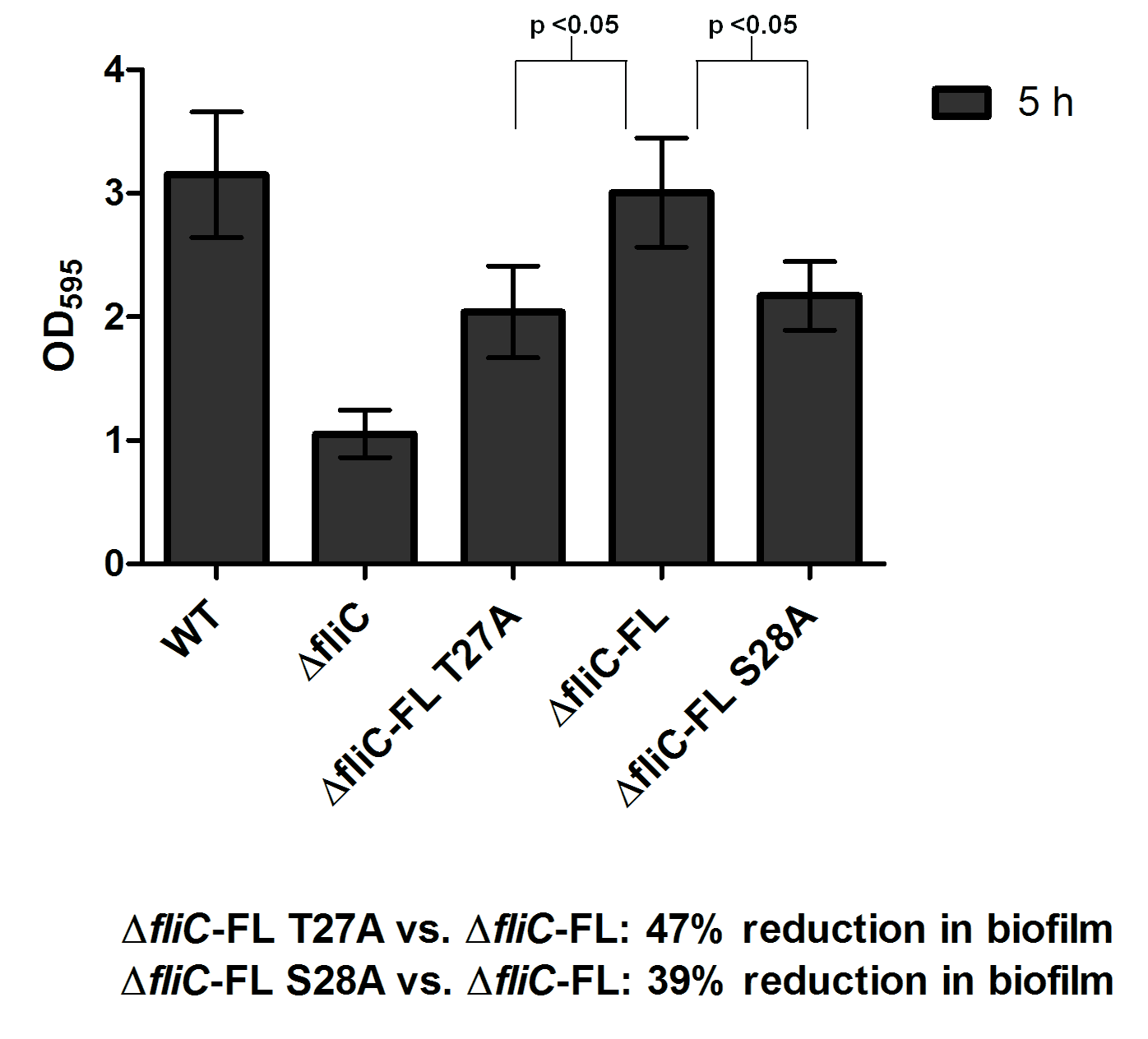

Supplement: S3 Fig — Quantification of biofilms formed in polystyrene round-bottom tubes by crystal violet staining at 5h for PAO1 WT, ΔfliC, ΔfliC-FL T27A, ΔfliC-FL and ΔfliC-FL S28A strains. Error bars indicate mean ± SD computed from four biological replicates with five technical replicates each. Student’s t-test p-values <0.05 for ΔfliC-FL T27A vs. ΔfliC-FL and ΔfliC-FL S28A vs. ΔfliC-FL. (TIF) [file pone.0164155.s003.tif]

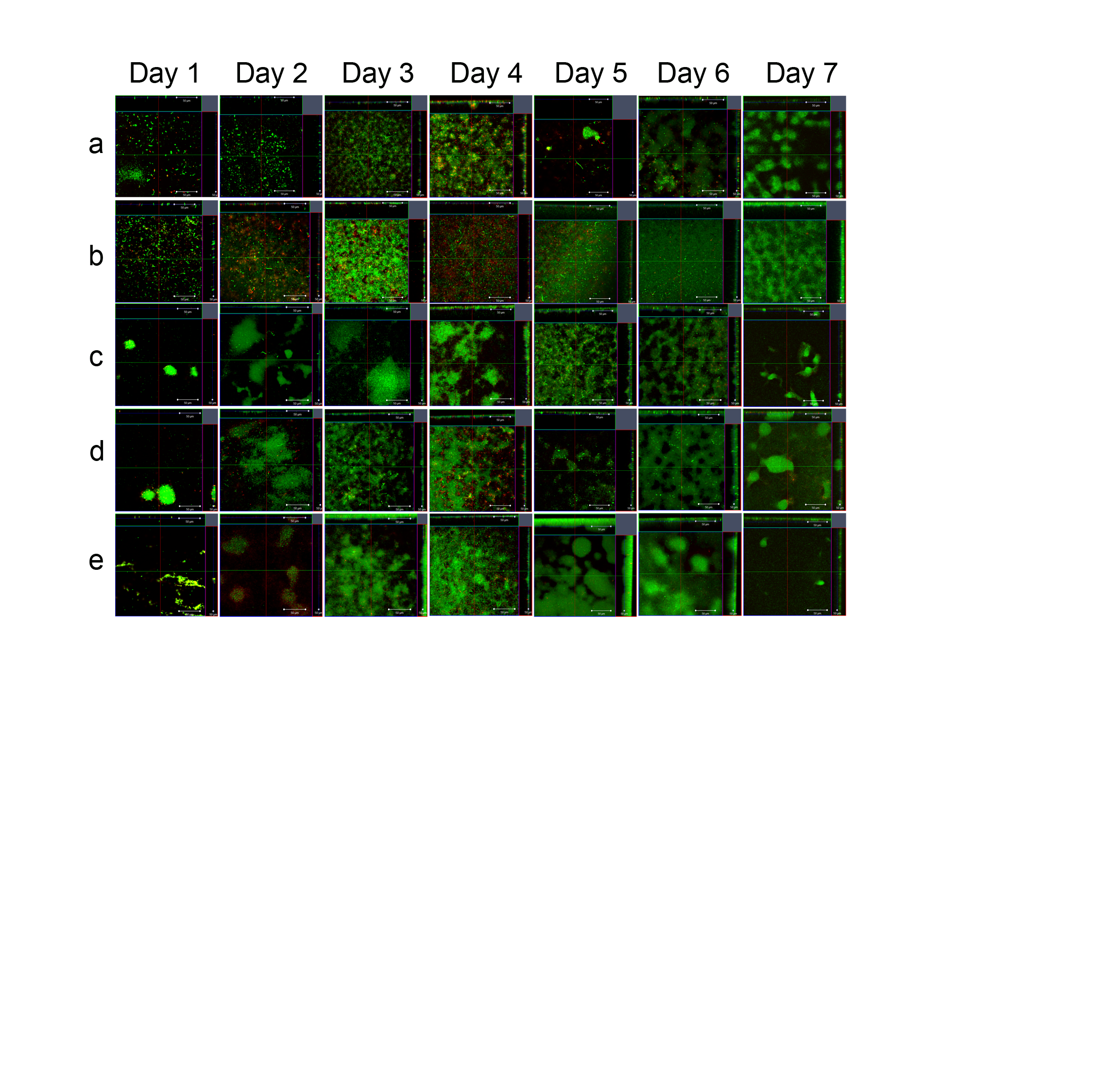

Supplement: S4 Fig — Comparison of biofilm architecture in confocal-ortho view for PAO1 WT, ΔfliC, ΔfliC-FL T27A, ΔfliC-FL and ΔfliC-FL S28A strains across all 7 days. Live and dead cells are represented in green and red, respectively. Panels are represented as a-WT, b-ΔfliC, c-ΔfliC- FL T27A, d-ΔfliC-FL and e-ΔfliC-FL S28A, respectively. Magnification is under 40X oil lens. Scale bars indicate a distance of 50 μm. (TIF) [file pone.0164155.s004.tif]

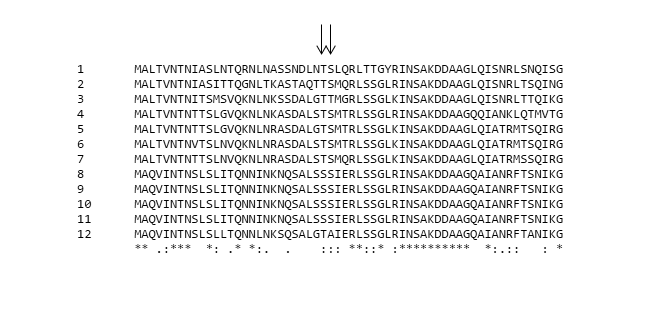

Supplement: S5 Fig — Clustal Omega alignment shows the extent of conservation of threonine 27 and serine 28 residues across different bacterial species. Species are indicated as 1- P. aeruginosa PAO1, 2- P. putida GB-1, 3- P. putida KT2440, 4- P. putida W619, 5- P. putida F1, 6- P. protegens Pf-5, 7- P. flectens Pf101, 8- E. coli K12, 9- E.coli K12 W3110, 10- E.coli CFT073, 11- E.coli EDL93 and 12- S. paratyphi A SARB42 respectively. Identical, strongly similar and weakly similar residue positions are indicated as (*), (:) and (.) respectively. (TIF) [file pone.0164155.s005.tif]
